# Does Environmental Instability Favor the Production and Horizontal Transmission of Knowledge regarding Medicinal Plants? A Study in Southeast Brazil

**DOI:** 10.1371/journal.pone.0126389

**Published:** 2015-05-20

**Authors:** Gustavo Taboada Soldati, Natália Hanazaki, Marta Crivos, Ulysses Paulino Albuquerque

**Affiliations:** 1 Universidade Federal de Juiz de Fora, Juiz de Fora, Minas Gerais, Brasil; 2 Laboratório de Ecologia Humana e Etnobotânica, Universidade Federal de Santa Catarina, Florianópolis, Santa Catarina, Brasil; 3 Laboratorio de Etnografía Aplicada, Universidad Nacional de La Plata, La Plata, Buenos Aires, Argentina; 4 Laboratório de Etnobiologia Aplicada e Teórica, Universidade Federal Rural de Pernambuco, Recife, Pernambuco, Brasil; Durham University, UNITED KINGDOM

## Abstract

Greater socio-environmental instability favors the individual production of knowledge because innovations are adapted to new circumstances. Furthermore, instability stimulates the horizontal transmission of knowledge because this mechanism disseminates adapted information. This study investigates the following hypothesis: Greater socio-environmental instability favors the production of knowledge (innovation) to adapt to new situations, and socio-environmental instability stimulates the horizontal transmission of knowledge, which is a mechanism that diffuses adapted information. In addition, the present study describes “how”, “when”, “from whom” and the “stimulus/context”, in which knowledge regarding medicinal plants is gained or transferred. Data were collected through semi-structured interviews from three groups that represented different levels of socio-environmental instability. Socio-environmental instability did not favor individual knowledge production or any cultural transmission modes, including vertical to horizontal, despite increasing the frequency of horizontal pathways. Vertical transmission was the most important knowledge transmission strategy in all of the groups in which mothers were the most common models (knowledge sources). Significantly, childhood was the most important learning stage, although learning also occurred throughout life. Direct teaching using language was notable as a knowledge transmission strategy. Illness was the main stimulus that triggered local learning. Learning modes about medicinal plants were influenced by the knowledge itself, particularly the dynamic uses of therapeutic resources.

## Introduction

Cultures can be understood as cybernetic systems with time and spatial dynamics similar to those of biological evolution [1]. Two processes are fundamental to cultural dynamics: the individual production of knowledge and transmission of this information [2,3,4]. Individual production is the process by which an individual gains, mainly by experimentation (with little or no social influence) [5], information that serves as a source of cultural variability [1]. Cultural transmission is a system of cumulative inheritance by which information and behaviors are transferred (within a social group) through interactions between pairs of individuals or with the products of the interaction [5, 6]. Transmission allows information to be disseminated, selected and fixed within a population [7].

The nature and frequency of individual production and the transmission of knowledge in a population are not random and are determined by environmental and social factors [3,8,9,10]. For instance, areas with highly variable environmental conditions favor a higher frequency of individual knowledge production. Although it is a more costly strategy, the new information produced will always be adapted to the new environmental conditions [3]. Copying in unpredictable environments can be disadvantageous because the information quickly becomes obsolete [3,5].

The process of cultural transmission is heterogeneous and can occur with different strategies of knowledge transmission [1,2,11]. Hewlett and Cavali-Sforza [8] categorized the transfer of information within a group in the following manners: a) vertical, between parental generations; b) horizontal, between non-parental pairs in the identical generation; c) one to many, when the models are leaders, local specialists or media and d) many to one, when older individuals serve as a reference for learning. Similar to individual production, the frequency of cultural transmission modes depends on the socio-environmental context. Stable environments favor vertical conservative transmission [9,10]. This pathway remains favored when the transmitted knowledge is highly adaptive and linked to the survival or fertility of individuals. Otherwise, the three alternative modes of transfer (less conservative and more propagative) are stimulated in changeable environments [9,10]. Therefore, the frequency at which the production and transmission of events occurs (and their different mechanisms) in a population may reflect adaptive strategies.

However, these ideas are the result of mathematical models and laboratory experiments. Studies on real systems are rarely conducted. As emphasized by Mesoudi [1], laboratory studies may attempt to reproduce natural systems but can never capture their complexity. Thus, there is a gap between the models and the actual knowledge systems.

Studies investigating the transmission of local ecological knowledge [8,10,12,13,14,15,16,17,18,19,20] provide models for investigating cultural evolution. Childhood is the most important phase for learning with parental role models being most important. In addition, learning also occurs during adulthood, but other modes of transmission are used [8,10,12,13,14,15,16,17,18,19,20]. Several investigations have confirmed that vertical transmission occurs more often between parents and children of the same sex, thus depending on the model's gender [8,10,15,17]. Moreover, the transmission modes depend on the nature and complexity of the knowledge [8]. Recent investigations, in addition to describing the transmission process, have tried to understand the process based on evolutionary logic (see [10,19,21,22,23]). For example, Reyes Garcia et al. [21] found that the models' prestige, an important factor in knowledge transmission, is minimally determined by plant knowledge and is not influenced by age. Heinrich and Broessh [22] concluded that the bias related to model selection, especially in adulthood, is the most important process in cultural evolution, surpassing the role of individual production and natural selection, which will have a lesser role in cultural variation.

In this study we used empirical data to test several theoretical assumptions that have been proposed in the literature based on mathematical models. Thus, the production and transmission of the local knowledge in three communities with distinct characteristics were investigated using an evolutionary approach. We use local knowledge of medicinal plants as the analytical unit by assuming that this knowledge includes a strong adaptive component and can be used as a model to reveal processes of cultural adaptation. The communities represented different levels of socio-environmental instability, i.e., security in the perpetuation of the lifestyles from the use of resources. We tested whether a) the production of new knowledge was favored in situations with high socio-environmental instability and b) vertical transmission was more frequent in stable environments. It was expected that the frequency of knowledge production (innovation) would be greater in environments with higher instability. Conversely, vertical transmission would be more prevalent in stable environments. Finally, we describe “how”, “when” and “in which context” individuals learn about medicinal plants. With empirical data, our findings show that cultural evolution demands greater complexity than current mathematical models provide to explain the spatial and temporal dynamics of cultural systems.

## Materials and Methods

### Study area

This study was conducted in a semiarid region in the north of Minas Gerais state, Brazil, where a lengthy annual drought is a limiting factor for agricultural activities [24]. The area is known as a 'vegetational mosaic', where characteristic formations of three large ecosystems merge: savanna, Atlantic rainforest and dry forest [24]. Due to its geographical location, the region has vegetation that is difficult to characterize; the vegetation displays complex floristic composition that is distributed in various vegetation types, which alternate according to the topography and soil conditions [25]. Adding to the composition of this ecotone region is a wide range of traditional identities such as indigenous, maroons, and other local cultural groups known as *sertanejos*, *catingueiros* and *vazanteiros*, which are all based on familiar agriculture, cattle and other animals and the extraction of natural resources.

The study was performed in the municipality of Capitão Enéas (16°42’08”S, 43°42’39”W), composed of a small urban center and a wide rural area with dispersed human settlements. The rural study area is dominated by "mata secas" (dry forests) [12] that can be organized into four vegetation types: 1) xerophytic formation in the limestone outcrops on mountain tops; 2) mata seca (deciduous seasonal mata seca) of limestone escarpments on the edge of mountains; 3) mata seca of colluvial slopes at the base of mountains; and 4) mata seca in marshland on oxisols. The most striking feature of the region is the strong seasonality, with seven dry months followed by months of uncertain rainfall.

Three communities with distinct socio-environmental characteristics were selected: a rural community, Bico da Pedra, and two communities of land reform, Darcy Ribeiro and Renascer (Table 1). Bico da Pedra (16°14’68” S, 43°68’38” W) consists of 80 individuals: 38 females and 42 males. There is a strong kinship system in the area (such as extended family), in which there is at least one next of kin in the community for all households. There are also consanguineous marriages (e.g., cousin marriages). The inhabitants maintain an enduring traditional relationship with their environment. The three heads of family who were not born locally have origins in a similar environmental context dominated by dry forest. Occupations are associated with agriculture, growing corn, beans and pumpkin and maintaining vegetable gardens. The home gardens of Bico da Pedra are old and offer a vast wealth of plant resources, notably medicinal plants, which is important for familial sovereignty [25]. The land reform community of Darcy Ribeiro (16°10’00” S, 43°69’89” W) is composed of 25 allotments that are inhabited by 27 families with 76 individuals, including 30 females and 46 males. The formation of this group was recent and the result of a national land reform policy. Group members arrived in Darcy Ribeiro as late as 2009 after much regional migration. Seven families have local origins, and twenty derived from distant regions. Of the latter families, ten migrated from locations dominated by dry forest, two migrated from the savanna and eight migrated from urban centers. Furthermore, only six families had close kinship between with one another. Therefore, for many families, contact with the current environmental context and between social pairs was fairly recent. Resources in home gardens are scant, which reduces a family’s ability to fulfill basic daily demands. Finally, Renascer (16°18’77” S, 43°65’44” W) is composed of 25 families and a total of 73 individuals (26 females and 47 males) with similar characteristics to Darcy Ribeiro. However, there was a greater level of regional migration events in Renascer and fewer local families were incorporated (only three). Moreover, there were more families from the savanna and urban centers (five and nine, respectively) and fewer consanguineous ties. Finally, political expression, or the ability to organize locally and nationally to claim and secure their rights, was weak compared to that of other locations.

**Table 1:** Social and environmental character used to fit the Bico da Pedra rural community and the land reform community Darcy Ribeiro and Renascer, Capitão Enéas, Brazil in a gradient of environmental and social stability.

Social group	Bico da Pedra Rural community	Darcy Ribeiro land reform community	Renascer land reform community
contact with the current environment and its plant resources	old	recent	recent
medical safety promoted by existing medical resources in backyards	high	low	low
formation time	old	recent	recent
the kinship system	high	low	very low
contact with social peers	old	recent	recent
collective events of regional migration	none	four	six
families of local origin (Dry Forest)	all	ten	three
families originating from Savanna	none	two	five
families originating from urban areas	none	eight	nine
political expression	high	high	low
**categorization of the socio-environmental stability situation**	**high**	**intermediate**	**low**

### The concept of socio-environmental instability

The differences among the three presented communities make it possible to recognize them as distinct stability states and constitute an ideal model to test in real systems, such as how the conditions in which a culture is inserted influence learning strategies. For this purpose, the current study imports the "environmental variability" concept (see [3,9,10]), defined simply as a situation in which “half of each cohort experiences environment 1 and the other half of each cohort experiences state 2”. In short, this concept emphasizes the temporal dynamic and special environmental conditions. Because this concept has only been used in mathematical modeling on cultural evolution, the current study makes its transposition to the investigated realities, thus encompassing, in addition to environmental conditions, social characteristics of the studied groups. To that end, the concept of socio-environmental stability considers a reality in which a culture does not experience the variations in environmental and social conditions in which they are inserted. Socio-environmental stability occurs when individuals from a group have the autonomy to provide for individual and family demands, thus reproducing their way of life by clinging to existing social relationships and utilizing elements from the local landscape.

The community of Bico da Pedra maintains a vast and detailed understanding of local resources and landscape dynamics, i.e., an ancestral knowledge, which is a result of the long period of contact with their environment. Similarly, the presence of developed agroforestry home gardens strengthens socio-environmental stability by guaranteeing that in situations of demand, medicinal resources can be quickly and easily accessed, thereby promoting greater health security. However, although the areas are well known to some of the families, constant migration in both settlements, Darcy Ribeiro and Renascer, results in individuals living in areas with no management systems. This study is based on the presumption that a family who developed their knowledge in an urban or savanna area will have difficulty accessing essential natural resources where dry forests dominate, i.e., the city of Capitão Enéas. Migration can affect the medical system, possibly leading to a loss of knowledge [26,27]. The low prevalence of kinship, the presence of individuals from different origins and cultural backgrounds, the high frequency of political divergence and the low political power to guarantee familial needs makes the two land reform communities a model of low social stability. In the Brazilian agrarian context, greater political power enables greater access to benefits and government programs, such as public health; therefore it is important to understand the social and environmental instability of the groups investigated. These variables (with the exception of political power) are diametrically opposite in the community of Bico da Pedra. In Darcy Ribeiro, these variables could be considered intermediate when compared to the other two communities. Therefore, it is assumed that the community of Bico da Pedra shows greater socio-environmental stability, Darcy Ribeiro displays an intermediate level of stability, and Renascer represents low socio-environmental stability (Table 1).

As part of the research sampling design, we aimed to include human groups who experienced the biggest social and environmental variation present in the investigated reality, assuming the complexity of the studied region. Two situations are presented in order to give a better understanding of the processes and assumptions investigated. Each is hypothetical and extreme with regard to the concept of socio-environmental instability. The situation of greater stability would be found in groups composed mainly of people who were born and lived in localities dominated by Dry Forests, who have strong family relationships. Migrant groups would represent the other end of this hypothetical socio-environmental variation. They would come from different phytogeographic contexts, including large urban centers. These groups experienced many migration events and do not have any family relationships with each other.

Because the current study uses local medical systems as a model to evaluate the relationship between socio-environmental stability and learning strategies, all of the characteristics that were used to categorize the three communities into one gradient were influenced, which is evidenced by the use and knowledge of medicinal resources.

### Collection of ethnobotanical data

Free lists of known medicinal resources were constructed with the heads of families (men and women) that were present during the approach. Out of 58 heads of families, there were 19 in Bico da Pedra (95% of the total possible informants), 19 in Darcy Ribeiro (71%) and 20 in Renascer (77%). The main objective of this free list was to construct a database for the selection of five medicinal plants used in the second section of the study. We opted to use a lower number of medicinal plants in the second interview because this stage was developed to understand the learning process and was therefore very extensive and tiring for the informant. The selection of these five plants was random, and this randomization considered only plants mentioned by each informant. This procedure was performed in the informant's absence, during the interval between the free list making and the semi-structured interview, using a formula “=RANDBETWEEN(<Bottom>,<Top>)” available in EXCEL software, version 2013.

With the five medicinal plant species that were selected by each informant, a semi-structured interview [28] was performed to evaluate the process of the individual production and transmission of knowledge. Most studies that evaluated the transmission of local knowledge did so from semi-structured interviews in which the informants were directly asked how the process of learning had occurred (see [14,15,16,17,29]). The informant’s report reflects the informant’s perception and may not reflect actual patterns of transmission if there is a degree of bias in the recall or in the social recognition of transmission episodes. It is believed that the answers are generalized and are biased to favor only parental models [30]. As a result, this methodology based on self-reported information is categorically criticized for valuing parents as models of learning (vertical routes) and may not be the most appropriate for studies of knowledge transmission (see [8,9,19,22,30]). In view of this criticism, several authors have advocated the use of methodologies that allow direct observation of transmission events, such as those based on ethnographic approaches [18,19,22].

The present study sought to remedy the shortcomings of studies based on self-reported information by adopting another approach involving the design of a more complex semi-structured interview based on explicit and detailed reports of the learning moments experienced by the informants. It is believed that this methodological option has accessed specific information, which is individualized, trusted and rich in details. This approach contributes to nullifying the weaknesses of interviews based on simple self-reported information by validating the interview as a tool for the study of the transmission of knowledge [31,32]. However, it is assumed that this approach does not completely solve the biases associated with self-reporting methodologies, and so this is a methodological limitation of this study.

In this sense, for each of the five selected medicinal plants, the following questions were asked: *“Do you recall how you learned about* (the name of the plant)? *Could you tell me about it?”* Based on the initial information, the informant was encouraged to relate *“when”* and “*from whom*” he/she learned the information and the “*context of learning*”, i.e., in what situation was the information produced or copied? This interview provided detailed information regarding the events leading to the production and transmission of knowledge. When an informant identified an event of knowledge production, the events that led to this innovation were ascertained, i.e., why the plant was chosen (as a therapeutic agent) and whether there were morphological or organoleptic characteristics guiding the thought process.

This study conforms to Brazilian legislation for research with traditional communities or human populations, was approved by the Ethics Committee of the Federal University of Viçosa (#014/2011). All informants that agreed to participate in this research provide a written consent by sign a document which explained the objectives and all procedures of this investigation. This consent procedure was also approved by Ethics Committee of the Federal University of Viçosa. As this study was carried out on a protected or private area, needed no specific permission from any authority. For future permissions the “Instituto Chico Mendes de Conservação da Biodiversidade” (ICMBio) of the “Ministério do Meio Ambiente” (MMA) should be contacted. Finally, the field studies did not involve endangered or protected species. The three social groups that participated in this research are located by the following coordinates: Bico da Pedra (-16°14’68”S, -43°68’38”W), Darcy Ribeiro (-16°10’00”S, -43°69’89”W) and Renascer (16°18’77’, -43°65’44”).

### Data analysis

The socio-environmental differences in stability among the three communities are treated as categorical (Table 1), with no measure of the relative scale of difference among the communities for this variable. The use of staggering, such as with an analysis factor, would make the analyses more robust by allowing the use of multinomial logistic regression. However, when considering the complexity of the study realities and the variables used to define the socio-environmental stability categories, we unfortunately do not have the ability to sort the three communities at the same scale. This impossibility is particularly caused by the absence of quantitative indicators on socio-environmental stability, which is why we opted for a categorical analysis that, even if less robust, allows us an approximation of the studied phenomenon.

The information obtained from the interviews was organized into four categories, as described in cultural evolution studies [2,5]: “from whom it was learned”, “modes of transmission”, “when it was learned” and “how it was acquired”. These categories evaluated when the knowledge was learned/produced, how the new knowledge was learned/produced, the learning model and the transmission type. The category “stimulus-context” was added based on the presumption that all knowledge is triggered by a stimulus [33]. With this category, we aimed to assess the main stimuli that trigger one’s learning about medicinal plants. When the informants reported that learning occurred from more than one individual, the initial source (the model) was determined, and only the primary model was considered for the analyses.

To test the first hypothesis (that individual knowledge production is favored in unstable environments), we compared the frequency of learning citations with events of knowledge transmission across the three communities (matrix: three by two) using the Chi-square test with Monte Carlo randomizations unilateral to correct the p value and account for values smaller than five. The “R” Program for Statistical Computing was used for this analysis. The identical statistical test was used to test the second hypothesis (that the vertical transmission of knowledge is stimulated in stable environments). We compared the four cultural transmission modes frequencies among the three communities (matrix: three by four). When significance was attained, Chi-square tests (matrix: two by two) were performed to determine which categories differed statistically.

The frequencies of the categories “when”, “how”, “from whom” and “stimulus-context” were compared among the communities using the G test in the Biostat program version 5.0 [34]. Similarly, when significant differences were present, 2 x 2 tests were performed using the Chi-square or G test (when any frequency was smaller than five).

## Results

### Is the individual production of knowledge favored in situations of socio-environmental instability?

The hypothesis that individual knowledge production of medicinal plants is stimulated in situations of socio-environmental instability was not supported by the data. This process had a low expression in the three social communities and was represented by only one report in Bico da Pedra and Darcy Ribeiro and four in Renascer (Table 2). These totals did not differ statistically among the communities (G = 12.07, DF = 1 and p = 0.0703). In Bico da Pedra, the reported innovation referred to a new therapeutic use from a medicinal species that was previously known locally. In the case of Darcy Ribeiro and Renascer, the cited innovations referred to the discovery of new medicinal resources.

**Table 2:** The category frequencies used to analyze the production and transmission of local knowledge about medicinal plants in the community of Bico da Pedra, Darcy Ribeiro and Renascer, Capitão Enéas, Minas Gerais, Brazil.

Category	BICO DA PEDRA	DARCY RIBEIRO	RENASCER
Learning strategies			
Transmission of knowledge	94 (a, A)	94 (a, A)	100 (a, A)
Individual production of knowledge	1 (a, B)	1 (a, B)	4 (a, B)
Strategies of knowledge transmission			
Vertical	73 (a, A)	73 (a, A)	69 (b, A)
Horizontal	15 (a, B)	17 (a, B)	31 (b, B)
Many-to-one	5 (a, C)	3 (a, C)	0
One-to-many	2 (a, C)	2 (a, C)	0
From whom is it learned?			
Mother	43 (a, A)	52(a, AB)	36 (a, B)
Father	9 (b, A)	13(b, AB)	22 (a, B)
Grandparents	9 (b, A)	6 (b, AB)	10 (b, B)
Friends or acquaintances	6 (b, A)	5(b, AB)	9 (b, B)
Neighbors	6 (b, A)	12(b, AB)	11 (b, B)
Uncles	6 (b, A)	3(c, AB)	1 (c, B)
When is it learned?			
Child	52 (a, A)	61 (b, A)	44 (c, A)
Adult	30 (a, B)	32 (b, B)	36 (c, A)
Adolescent	13 (a, C)	2 (b, C)	20 (c, B)
How is it learned?			
Teaching	85 (a, A)	83 (a, A)	88 (a, A)
Observation	9 (a B)	11 (a, B)	7 (a, B)
Individual experiences	1 (a, C)	1 (a, C)	5 (a, C)
Stimulus—context			
Event of illness or necessity	80 (a, A)	87 (a, A)	89 (a, A)
Exposure to resource	7 (a, B)	4 (a, B)	6 (a, B)
Conversations about medicinal plants	8 (a, B)	3 (a, B)	5 (a, B)
Courses	0	1 (a, C)	0

The frequencies correspond to the sum of learning events for each informant in each community. The lower case letters that follow the frequencies represent the results of statistical comparisons among the communities. Different letters in the same row express a significant difference among the communities. The capital letters correspond to the results of the categories within each community. Different letters in the same column express a significant difference within the community.

The cultural transmission was the most important learning process in the three locations (x^2^_BICO DA PEDRA_ = 13.30, DF = 1 and p = 0.001; x^2^_DARCY RIBEIRO_ = 10.12, DF = 1 and p = 0.002; x^2^_RENASER_ = 14.57, DF = 1 and p = 0.001). Learning occurred by cultural transmission in 115 citations in Bico da Pedra (99.3%), 102 in Darcy Ribeiro (99.1%) and 102 in Renascer (96.2%). Altogether, 23 learning models (that were not necessarily represented in all three communities) were detected. Mothers were the primary source of knowledge followed by fathers (Table 2). In both communities, neighbors were also influential as models but differed statistically from fathers (x^2^_DARCY RIBEIRO_ = 30.26, DF = 2 and p < 0.005 and x^2^_RENASCER_ = 28.35, DF = 2 and p < 0.0043). Although less frequent, the following learning models were also reported: “older people”, “boss”, “mother-in-law”, “father-in-law”, “people”, “unknown people”, “son-in-law”, “godfather” or “godmother”, “grandmother”, “wife” or “husband”, “sister” or “brother”, “media”, “cousins”, “professionals of traditional medicine”, “great grandparents”, “sister-in-law” or “brother-in-law” and “healthcare professionals”. Notably, many of the citations generalized the learning model (source), i.e., “older people” referred to elderly individuals in general, and “people” referred to locals in general. The learning model was only revealed when the specific source of knowledge was questioned. Knowledge of a medicinal resource could originate from more than one model. From the resources analyzed, 50 plants in Bico da Pedra, 45 in Darcy Ribeiro and 47 in Renascer were copied from more than one individual.

### Is horizontal transmission favored in situations of socio-environmental instability?

The hypothesis that vertical transmission is favored in situations of greater environmental stability was not supported by the data. In Bico da Pedra and Darcy Ribeiro, four knowledge transmission pathways were identified, vertical, horizontal, many-to-one and one-to-many, whereas only two, vertical and horizontal, were identified in Renascer. Vertical transmission was the most important method of transference in all communities (x^2^_BICO DA PEDRA_ = 33.03, DF = 1 and p < 0.0001; x^2^_DARCY RIBEIRO_ = 30.99, DF = 1 and p<0.0001; and x^2^_RENASCER_ = 12.81, DF = 1 and p < 0.0004). The diffusion of knowledge (horizontal transmission) was the second most frequent pathway among the three communities and differed statistically from the other processes (x^2^_BICO DA PEDRA_ = 3.33, DF = 1 and p < 0.05; x^2^_DARCY RIBEIRO_ = 9.38, DF = 1 and p < 0.01; x^2^_RENASCER_ = 12.16, DF = 1 and p < 0.01) (Table 2). Thus, the three communities displayed the identical dynamic of knowledge transmission with vertical (parental models) transmission being the most important followed by horizontal transmission. However, when comparing the cultural transmission modes frequencies among the communities, Bico da Pedra did not differ from Darcy Ribeiro (G = 4.21, DF = 3 and p < 0.2554), but these two groups differed from Renascer with G = 15.29, p < 0.0001, DF = 3 and G = 8.23, DF = 3 p < 0.0161, respectively. Therefore, despite vertical transmission being the most important, the frequency of horizontal transmission (diffusion processes) was greater in Renascer relative to the other communities.

### When was the knowledge learned?

For all communities investigated, childhood was the most frequently cited period for learning the properties of medicinal plants. However, only Bico da Pedra (x^2^ = 5.589, DF = 2 and p = 0.0179) and Darcy Ribeiro (x^2^ = 9.045, DF = 2 and p = 0.0023) showed significant differences (Table 2). The distribution of citations (regarding the time of learning), when compared two by two, differed between all communities (G_BICO DA PEDRA x DARCY RIBEIRO_ = 10.2064, DF = 2 and p = 0.0059; G_BICO DA PEDRA x RENASCER_ = 2.3997, DF = 2 and p = 0.3011; G_DARCY RIBEIRO x RENASCER_ = 20.32, DF = 2 and p < 0.0001). The largest difference was observed between Darcy Ribeiro and Renascer. For the latter land reform community, learning during adolescence was significantly higher. This category also explained the difference between Bico da Pedra and Darcy Ribeiro (Table 2). In four instances in Bico da Pedra, four in Darcy Ribeiro and three in Renascer, the current knowledge regarding a medicinal resource was obtained in more than one life stage. In these cases, the informant learned about the identical resource from distinct events.

### How was the knowledge learned?

The transmission of knowledge in the three communities occurred through two processes: teaching and observation. Eight subcategories of teaching, the most important process in the three communities, were recognized (G_BICO DA PEDRA_ = 59.73, DF = 1 and p = 0.0001; G_DARCY RIBEIRO_ = 79.45, DF = 1 and p = 0.0001; and G_RENASCER_ = 69.79, DF = 1 and p = 0.0001) with “explanation or indication” being the most important subcategory in all the settlements (x^2^_BICO DA PEDRA_ = 21.20, DF = 1 and p < 0.0001; x^2^_DARCY RIBEIRO_ = 6.041, DF = 1 and p < 0.013; x^2^_RENASCER_ = 30.947, DF = 1 and p < 0.0001) followed by “prepared the medicine for the person” (x^2^_BICO DA PEDRA_ = 18.15, DF = 1 and p < 0.0001; x^2^_DARCY RIBEIRO_ = 8.26, DF = 1 and p < 0.0198; x^2^_RENASCER_ = 32.46, DF = 1 and p < 0.0001).

Three types of observation were recorded: “preparing medicine for other people”, “seeing people collecting the resource” and “people asking for medicine for a person”. “Preparing medicine for other people” was the most important category in the three groups (x^2^_BICO DA PEDRA_ = 30.788, DF = 2 and p< 0.0001; x^2^_DARCY RIBEIRO_ = 20.965, DF = 2 and p< 0.0198 and x^2^_RENASCER_ = 32.672, DF = 2 and p< 0.0001), whereas the last two observation types did not differ statistically.

### Stimulus/context

Illness events, individual or in others, lived or shared, were the most important stimuli/context for learning medicinal plants in each group (x^2^_BICO DA PEDRA_ = 169.468, DF = 2 and p< 0.0001; x^2^_DARCY RIBEIRO_ = 266.310, DF = 2 and p< 0.0001; x^2^_RENASCER_ = 242.00, DF = 2 and p< 0.0001) (Table 2). When analyzed in detail, personal illness (individual) received the highest number of references. However, only in Bico da Pedra, this category did not differ from illness in the family (x^2^ = 1.929, DF = 1 and p = 0.1650) or of a non-family member (x^2^ = 3.449, DF = 1 and p = 0.1350) (Table 2). There was no difference between the citation distributions among the three communities (G = 11.6366; p = 0.0689).

The second most important stimuli/context that favored local learning was “exposure to the resource” and “conversations about medicinal plants”. These categories were not significantly different in the three communities (x^2^_BICO DA PEDRA_ = 169.468, DF = 1 and p< 0.0001; x^2^_DARCY RIBEIRO_ = 266.309, DF = 1 and p< 0.0001; x^2^_RENASCER_ = 244.00, DF = 1 and p< 0.0001). In Darcy Ribeiro, the knowledge regarding a specific plant resource was favored by a training course in the area. These training courses are common in land reform areas.

## Discussion

### Is the individual production of knowledge favored in situations of socio-environmental instability?

Individual knowledge production was less prevalent in the three groups than information learned by cultural transmission. In addition, the proportion of these two learning strategies did not differ among the groups. Therefore, the hypothesis that the “individual production of knowledge events are stimulated in unstable environments” was not supported. We expected that this learning process would have been favored in group with greater socio-environmental instability. Darcy Ribeiro included seven families that had previously lived in the region, providing this community with a greater familiarity of their local environment (dry forest), which might favor social transmission to the detriment of the production of new knowledge. Renascer is a community formed almost exclusively by individuals unknown to one another who originate from different regions and have migrated to settle in the current location. However, this result was not observed. In the three communities, resources whose properties were validated by conspecifics or experience were used. The low frequency of innovation events in the three communities can be explained by the dynamics of using medicinal plants. Because knowledge was directly linked to the survival of individuals, it promoted the use of previously known and validated resources over the expense of building new information. In the three groups, it is believed that there is little room for experimenting with new knowledge because the use of medicinal plants is based on past trust.

If innovation is not significant in the three communities investigated, what is the origin of the variability of information, which is an important process in cultural evolution [35]? The diversity contained in the current knowledge of these groups could be due to other processes, i.e., the unreliable transmission of knowledge, which is a process similar to biological mutation. Furthermore, other processes can promote knowledge variation such as the insertion of exotic information, i.e., regional migration and learning from external sources (radio, books and television). Leonti et al. [36] reported that written material on medicinal plants (books, notebooks or simple leaflets) influences local pharmacopoeias. Leonti and Casu et al. [37] and Ndhlala et al. [38] noted that globalization intensified exchanges between different medical systems (via media, television and the Internet). These methods considered the geographical origin of the information and whether it was native or exotic to the situation investigated. Future studies should extend beyond typical questions of “how”, “when” and “from whom” learning occurs and investigate the geographical origin of the information.

Regarding knowledge transmission, the main models (sources of knowledge) were family members, particularly mothers. This particular role of mothers reinforces, as previously noted, the transmission of knowledge and is dependent on the information content. Various studies have reported the role of mothers in areas including horticulture [16], wild foods [39,40] and medicinal resources [15]. However, it is believed that the mother's most important role as a learning model is in healing and maintaining family health.

### Is horizontal transmission favored in situations of socio-environmental instability?

The most important mode of cultural transmission in the three communities was “vertical”. Therefore, the hypothesis that the events involved in horizontal transmission of knowledge are stimulated in unstable environments was not supported. Vertical transmission was expected to be more abundant in Bico da Pedra relative to the other communities, which was not the case. Boyd et al. [41] reported that depending on the nature of the knowledge transferred, cultural mechanisms, such as ethnocentrism, discrimination or an intricate social organization, could favor vertical transmission. Durham [42] attributed these cohesive processes to “mechanisms of isolated transmission”. For example, Collard and Tehrani [43] demonstrated that the nature of textile knowledge in Turkmen cultures stimulates vertical transmission because the information is orally relayed during the manufacturing process. Mechanisms can act on an individual level, thus favoring the vertical transmission of knowledge of local plants. In the three communities investigated, these mechanisms were represented by the informants' confidence in using a given resource. Generally, this confidence was associated with illness experienced during childhood. Although the informants have learned new therapeutic resources as adults, the knowledge learned from parents was typically used and transmitted. Despite the informants having learned new therapeutic resources as adults, they use and consequent transmit knowledge of plants that have been learned from parents.

According to Ellen and Fisher [44], some cultural domains change more than others, making certain elements (traces) show some temporal and spatial stability. The presented data suggest, therefore, a cognitive system with low change rates. However, it does not indicate that the local medical practices are immutable or that there is a high fidelity in knowledge transmission in the investigated locations. The field data suggest that learning is not a passive process but instead a more complex and interactive process [44] in which the information is constantly reassessed and reconstructed. Aside from the low cultural alterations rate, the high frequency of the vertical transmission path suggests that cultural systems are heterogeneous and involve high variation in individual knowledge [8]. Moreover, the existing social differences among the three studied groups suggest, as shown by Tehrani and Collard [45], a regional scenario in which the social groups demonstrate a low homogeneity on medicinal plant knowledge.

The present study observed that vertical transmission was most important for transferring local knowledge [10,19,20]. Furthermore, vertical transmission repeats itself even in contexts that might favor horizontal transmission. Nevertheless, these studies suggested that horizontal transmission was favored in unstable environments, not that this mode became the most important. This result was observed in the present study, although vertical transmission was most important, Renascer (the community with greater environmental variation and a new social structure) also displayed the highest frequency of horizontal learning. As stated, the two hypotheses tested in this study were not supported. However, these results must be qualified in two ways. First, it is possible that situations of variability act on specific cognitive domains and, thus, with respect to the acquisition and transfer mechanisms (see [23]). However, it is believed that the knowledge of medicinal plants is a suggestive domain for assessing the role of the environment in transmission routes because this knowledge is directly linked to the survival of the individual and the social group. Finally, it is possible that the levels of socioenvironmental instability analyzed were not sufficiently broad to encourage individual production and horizontal transmission. In highly stable environments, vertical learning is favored because genetic predispositions (instincts) are the best "cues" for behavior. In moderately variable environments, the vertical pathways are stimulated. At the other end of this spectrum, i.e., in a situation of high variation, individual production is the most profitable strategy. It is possible that the socio and environmental conditions analyzed fit the center of this spectrum, not addressing situations that favor horizontal transmission or individual production. Therefore, it is suggested that future studies consider larger amplitudes in the cases of instability selected for analysis.

### When was the knowledge learned?

This study corroborates previous investigations (attempting to comprehend the transmission of local knowledge) (see [10,15,16,30,46,47]) and also recognized that childhood is a critical moment for learning. What is the explanation for this pattern? Demps et al [23] analyzed the transmission studies and found that the nature of knowledge determines a pattern of learning, headed by the authors as "age structured". According to these authors, the concrete knowledge, goals capable of verbalization, such as the name of the medicinal resources, are preferentially transmitted in childhood. As a result, it is expected that children are proficient in this cognitive domain before the age of twelve, as verified by Zager [48]. Otherwise, tacit knowledge and complex skills that demand, besides verbalization, exhibition and experimentation are transmitted later (see [49]). Therefore, the concrete and verbal nature of the names of medicinal resources advance their learning for children.

However, there is another evolutionary explanation. Nairne et al. [50] and Pandeirada [51] began with the assumption that information forms basic elements for living in a social group to describe an adaptive bias in the transmission of knowledge, thus suggesting that knowledge directly related to survival is more easily stored and transmitted. For example, while evaluating groups in the Congo, Hewlett et al. [19] confirmed that children preferably learn information about subsistence and essential skills. It is possible that information associated with survival, such as the use of medicinal plants, is learned in childhood as a block of basic knowledge for individual and social perpetuation. Thus, it is argued that childhood, as a preferential learning stage, has an adaptive explanation.

Learning also occurs in adulthood with approximately 30 to 35% of events recorded in this phase in the studied communities. It is hypothesized that learning at a later life stage occurs for the following reasons: a) the occurrence of diseases in the elderly, such as cancer, arthritis and back problems and b) lifestyle alterations, such as parenthood, which necessitate learning about medicinal plants.

### How was the knowledge learned?

Some studies have demonstrated that although parents, grandparents or local specialists represent important knowledge sources, one of the most important forms of learning is through trial and error [10,13,15,47,52]. The individual production of knowledge was not a common occurrence in the three communities. We hypothesized that experimentation with medicinal plants was not favored because current knowledge is linked to the survival of individuals. This knowledge is subject to different selective processes [53, 54] and is strongly fixed in the population. The present study does not refute that trial and error is important for the medical system, rather that is not responsible for producing frequent innovations.

The two processes of social transmission of information that were recorded in the three communities operate from different cognitive mechanisms [55]. For observation, an apprentice absorbs knowledge indirectly without the use of language. For teaching, behavior is modified to support learning [55] and enables the transmission of knowledge of complex or subjective skills in a more reliable manner [56]. Therefore, the greater frequency of teaching in the investigated contexts reflects a cultural adaptation as the use of medicinal plants is directly linked to biological survival, therefore demanding reliable transmission.

### Stimulus/context

Illness, particularly individual illness, was the most important stimulus for triggering the learning of medicinal plants in the three communities. According to Mathez-Stiefel and Vandebroek [20], the learning of medicinal plants in two Andean communities was a consequence of illness in the nuclear family. In these two communities, when an individual becomes ill, the immediate family members diagnose the illness, select the medicinal plant and recruit children to collect it [20]. Because transmission and learning depend on the production of knowledge, these processes are also influenced by context. Illness precipitates transmission and the learning of local plants, i.e., following an illness, medicine is discussed and prepared by the family members.

Thus, the main stimulus for learning about a medicinal resource is to fall ill or experience an illness within the nuclear family. A relationship may exist between the evolution of medicinal plant knowledge and illness episodes. There is a local knowledge of plants to banish or minimize a stimulus (illness) that selects this cognitive system, therefore building a relationship of “conflictive dependency”.

Episodes not only determine learning but all the nosological categorization designed by individuals. Acting on knowledge associated with healing is dependent on context. For example, Crivos [31,32] reported that information provided by an informant varies over time, and that this variation is dependent on the socio-environmental context. According to Crivos [32], informants attribute meaning and values to nosological categories connected to plants and their therapeutic value to utilize them in specific healing situations. Moreover, Crivos [32] claim that this conceptual domain is defined by the experiences in which these categories update themselves. Thus, the episode presents itself as an adequate context to reconsider the principles and criteria that rule local categorization processes for both actual diseases and their associated categories [31,32].

However, studies show that the knowledge of medical resources and health status are associated variables [57]. The same is the case for nutritional status [21]. How can knowledge of plants be stimulated by events of illness but associated with greater health? This contradiction can be resolved in terms of the complex process of illness and healing. A person can be healthy by not being sick or by rapidly healing. Based on the results of this study, it is expected that only the second situation will result in an extensive knowledge of plants combined with high health status. In this context, the variables used by McDade et al. [57] to indicate immune status reflect this second health situation more than the first one.

## References

[1] Mesoudi A. Cultural Evolution: how darwinian theory can explain human culture, synthesize the social sciences. 1st ed. Chicago: The University of Chicago Press; 2011.

[2] Cavalli-Sforza LL, Feldman M. Cultural transmission, evolution: a quantitative approach. 1st ed. Princeton: Princeton University Press; 1981 7300842

[3] Boyd R, Richerson PJ. The origins, evolution of human culture. Oxford: Oxford University Press; 2005.

[4] Mesoudi A, Whiten A, Lal K. Towards a unified science of cultural Evolution. J Behav Brain Sci. 2006; 29: 329–383. 17094820 10.1017/S0140525X06009083

[5] Lal KN. Social learning strategies. 2004; Learn Behav 32: 4–14. 15161136 10.3758/bf03196002

[6] Hoppitt W, Lal KN. Social processes influencing learning in animals: a review of the evidence. Adv Study Behav. 2008; 38: 105–165.

[7] Mesoudi A, Whiten A, Lal K. Perspective: is human cultural evolution darwinian? Evidence reviewed from the perspective of The Origin of Species. Evolution. 2004; 58: 1–11. 15058714 10.1111/j.0014-3820.2004.tb01568.x

[8] Hewlett BS, Cavalli-Sforza LL. Cultural transmission among Aka Pygmies. Am Anthropol. 1986; 88: 922–934.

[9] McElreath R, Strimling P. When natural selection favors imitation of parents. Curr Anthropol. 2008; 49: 307–316.

[10] Reyes-Garcia V, Molina JL, Broesch J, Calvet L, Fuentes-Pelaez N, McDade TW, et al Cultural transmission of ethnobotanical knowledge and skills: an empirical analysis from an amerindian society. Evol Hum Behav. 2009; 30: 1–12. 20046209

[11] Boyd R, Richerson PJ. Culture and the Evolutionary Process. Chicago. University Of Chicago Press; 1988.

[12] Ohmagari K, Berkes F. Transmission of indigenous knowledge and bush skills among the Western James Bay Cree women of subartic Canada. Hum Ecol. 1997; 25: 197–222.

[13] Zent S. Acculturation and ethnobotanical knowledge loss among the Piaroa of Venezuela: demonstration of a quantitative method for the empirical study of TEK change In: Maffi L editor. On Biocultural Diversity: linking language knowledge and the environment. Washington, London: Smithsonian Institution Press; 2001 pp. 190–205.

[14] Ladio A, Lozada M. Patterns of use and knowledge of wild edible plants in distinct ecological environments: a case study of a Mapuche community from northwestern Patagonia. Biodivers Conserv. 2004; 13: 1153–1173.

[15] Lozada M, Ladio A, Weig M. Cultural transmission of ethnobotanical knowledge in a rural Ccmmunity of northwestern Patagonia Argentina. Econ Bot. 2006; 60: 374–385.

[16] Eyssartier C, Ladio AH, Lozada M. Cultural Transmission of Traditional Knowledge in two populations of North-western Patagonia. J Ethnobiol Ethnomed. 2008; 4: 25–33. 10.1186/1746-4269-4-25 19077315 PMC2614966

[17] Tehrani JJ, Collard M. On the relationship between interindividual cultural transmission and population-level cultural diversity: a case study of weaving in Iranian tribal populations. Evol Hum Behav. 2009; 30: 286–300.

[18] Wyndham FS. Environments of Learning: Rarámuri Children’s Plant Knowledge and Experience of Schooling Family and landscapes in the Sierra Tarahumara Mexico. Hum Ecol. 2009; 38: 87–99.

[19] Hewlett BS, Fouts HN, Boyette AH, Hewlett BL. Social Learning among Congo basin hunter-gatherers. Philos Trans R Soc Lond B Biol Sci. 2011; 366: 1168–1178. 10.1098/rstb.2010.0373 21357239 PMC3049106

[20] Mathez-Stiefel SL, Vandebroek I. Distribution and transmission of medicinal plant knowledge in the Andean highlands: A case study from Peru and Bolivia. Evid Based Complement Alternat Med. 2012; 1–18. 10.1155/2012/959285 PMC3235884 22203885

[21] Reyes-Garcia V, Molina JL, Broesch J, Calvet L, Huanca T, Saus J, et. al Do the aged and knowledgeable men enjoy more prestige? A test of predictions from the prestige-bias model of cultural transmission. Evol Hum Behav. 2008; 29: 275–281.

[22] Heinrich J, Broessh J. On the nature of cultural transmission networks: evidence from Fijian villages for adaptive learning biases. Philos Trans R Soc Lond B Biol Sci. 2011; 1567:1139–1148. 10.1098/rstb.2010.0323 21357236 PMC3049092

[23] Demps K, Zorondo-Rodríguez F, García C, Reyes-García V. Social learning across the life cycle: cultural knowledge acquisition for honey collection among the Jenu Kuruba, India. Evol Hum Behav. 2012; 33: 460–470.

[24] Ab’Sáber AN Os domínios da natureza no Brasil: potencialidades paisagísticas. São Paulo: Ateliê Editorial; 2003 324p.

[25] Duque-Brasil R, Soldati GT, Costa FV, Marcatti AA, Reis-Jr R, Coelho FMG. Riqueza de plantas e estrutura de quintais familiares no semi-árido norte mineiro. Rev Bras Biociências. 2007; 5: 864–866.

[26] Pieroni A, Vandebroek I. Travelling cultures and plants: the ethnobiology and ethnopharmacy of migrations Studies in Environmental Anthropology, Ethnobiology, vol 7 New York: Berghahn Books; 2007.

[27] Medeiros PM, Soldati GT, Alencar NL, Vandebrok I, Pieroni A, Hanazaki N, et. al The use of medicinal plants by migrant people: adaptation maintenance and replacement. Evid Based Complement Alternat Med. 2012: 1–11. 10.1155/2012/807452 PMC3216396 22110548

[28] Albuquerque UP, Lucena RFP, Lins Neto EMF (2013) Selection of research participants In: Albuquerque UP, Lucena RFP, Cunha LVFC, Alves RRN. Methodos and techiniques in ethobiology and ethnoecology. New York: Springer pp. 1–13.

[29] Arco MPO, Torre-Cuadros MA, Reynel C, Infierno and Sonene Comunities Cultural transmission on Palms among Ese Eja communities in Peru. Biorem Biod and Bioavailab. 2011; 5: 92–99.

[30] Aunger R. The life history of culture learning in a face-to-face society. Ethos. 2000; 28: 445–481.

[31] Crivos M. Contribución al estudio antropológico de la medicina tradicional de los Valles calchaquíes (Salta Argentina) Tesis de Postgrado. Universidad Nacional de La Plata 2004

[32] Crivos M. El estudio de la narrativa de casos: una propuesta para el abordaje etnográfico de las alternativas médicas In: Idoyaga MA, editor. Los caminos terapéuticos y los rostros de la diversidad. La selección y combinación de medicinas en Argentina. Buenos Aires: CAEA-IUNA; 2001 pp. 87–113.

[33] Heyes CM. Social learning in animals: categories, mechanisms. Biol Rev. 1994; 69: 207–231. 8054445 10.1111/j.1469-185x.1994.tb01506.x

[34] Zar J H. Biostatistical Analysis,5th edition Pearson; 2009.

[35] Mesoudi A. An experimental simulation of the “copy-successful-individuals” cultural learning strategy: adaptive landscapes, producer—scrounger dynamics, and informational access costs. Evol Hum Behav. 2008; 29: 350–363.

[36] Leonti M. The future is written: impact of scripts on the cognition selection knowledge and transmission of medicinal plant use and its implications for ethnobotany and ethnopharmacology. J Ethnopharmacol. 2011; 134: 542–555. 10.1016/j.jep.2011.01.017 21255636

[37] Leonti M, Casu L. Traditional medicines and globalization: current and future perspectives in ethnopharmacology. Front Pharmacol. 2013; 4: 92–105. 10.3389/fphar.2013.00092 23898296 PMC3722488

[38] Ndhlala AR, Stafford GI, Finnie JF, Van Staden J. Commercial herbal preparations in KwaZulu-Natal South Africa: The urban face of traditional medicine. S. Afr. J. Bot. 2011; 77: 830–843.

[39] Setalaphruk C, Price LL. Children's traditional ecological knowledge of wild food resources: a case study in a rural village in Northeast Thailand. J Ethnobiol Ethnomed. 2007 3: 33–44. 17937791 10.1186/1746-4269-3-33 PMC2100045

[40] Garcia GSC. The mother—child nexus: knowledge and valuation of wild food plants in Wayanad Western Ghats India. J Ethnobiol Ethnomed. 2006; 2: 1–6. 16968535 10.1186/1746-4269-2-39 PMC1613234

[41] Boyd R, Bogerhohh-Mulder M, Durham W, Richerson PJ. Are cultural phylogenies possible? In: Weingart P, Richerson PJ, Mitchell SD, Maasen S, editors. Human by nature between biology and the social sciences. Mahwah: Lawrence Erlbaum Associates; 1996 pp. 355–386.

[42] Durham WH. Advances in evolutionary culture theory. Annu Rev Anthropol. 1992; 19: 187–210.

[43] Collard M, Tehrani J. Phylogenesis versus ethnogenesis in Turkmen cultural evolution In: Mace R, Holden CJ, Shennan S, editors. The evolution of cultural diversity: a phylogenetic approach. Walnut Creek: Leaf Coast Press; 2005 pp 110–132.

[44] Ellen R, Fischer MD. Introduction: on the concept of cultural transmission In: Ellen R, Lycett JL, Johns SE. Understanding cultural transmission in Anthropology: a critical synthesis. New York: Berghan Books; 2013 pp. 1–54.

[45] Tehrani JJ, Collard M. Do transmission isolating mechanisms (TRIMS) influence cultural evolution? Evidence from patterns of textile diversity within and between Iranian Tribal Groups In: Ellen R, Lycett JL, Johns SE. Understanding cultural transmission in Anthropology: a critical synthesis. New York: Berghan Books; 2013 pp. 148–164.

[46] Zarger RK, Stepp JR. Persistence of botanical knowledge among Tzeltal Maya children. Curr Anthropol. 2004; 45: 413–419.

[47] Srithi K, Balslevb H, Wangpakapattanawonga P, Srisangac P, Trisonth C. Medicinal plant knowledge and its erosion among the Mien (Yao) in northern Thailand. J Ethnopharm. 2009; 123: 335–342. 10.1016/j.jep.2009.02.035 19429381

[48] Zager RK. Acquisition and Transmission of Subsistence Knowledge by Q'eqchi' Maya in Belize In: Stepp JR, Wyndham FS, Zarger RK, editors. Ethnobiology ana cultural biodiversity. Athens, University of Georgia Press; 2002 pp. 593–613.

[49] Gurven M, Kaplan H, Gutierrez M. How long does it take to become a proficient hunter? Implications for the evolution of delayed growth. J Hum Evol. 2006; 51: 454–470. 16797055 10.1016/j.jhevol.2006.05.003

[50] Nairne JS, Pandeirada JNS, Thompson SR. Adaptive memory: the comparative value of survival processing. Psychological Science. 2008; 19: 176–180. 10.1111/j.1467-9280.2008.02064.x 18271866

[51] Nairne JS, Pandeirada JNS. Adaptive memory: is survival processing special? J Mem Lang. 2008; 59: 377–385.

[52] Frazão-Moreira A. Meninos entre árvores e lianas—aprendizagem do mundo e das plantas pelas crianças Nalu (Guiné-Bissau). Educação, Sociedade e Culturas. 1997; 7: 75–108. 10.5056/jnm14040 25344995

[53] Takahasi K Evolution of transmission bias in cultural inheritance. J Theor Biol. 1998; 190: 147–159. 9538463 10.1006/jtbi.1997.0541

[54] Holden CJ, Shennan S. Introduction to part I: tree-like is cultural evolution? In: Mace R, Holden CJ, Shennan S, editors. The evolution of cultural diversity: a phylogenetic approach. Walnut Creek: Leaf Coast Press; 2005 pp. 13–25.

[55] Heyes CM. Social learning in animals: categories and mechanisms. Biol Rev. 1994; 69: 207–231. 8054445 10.1111/j.1469-185x.1994.tb01506.x

[56] Csibra G, Gergely G. Social learning and social cognition: The case for pedagogy IN Johnson MH, Munakata Y, editors. Processes of change in brain and cognitive development. Attention and performance XXI. Oxford: Oxford University Press; 2006 pp. 249–274.

[57] McDade TW, Reyes-García V, Blackinton P, Tanner S, Huanca T, Leonard WR. Ethnobotanical knowledge is associated with indices of child health in the Bolivian Amazon. Proceedings of the National Academy of Science. 2007; 104: 6134–6139. 17389376 10.1073/pnas.0609123104 PMC1851091

